# Human milk microbiota profiles in relation to birthing method, gestation and infant gender

**DOI:** 10.1186/s40168-015-0145-y

**Published:** 2016-01-06

**Authors:** Camilla Urbaniak, Michelle Angelini, Gregory B. Gloor, Gregor Reid

**Affiliations:** Lawson Health Research Institute, 268 Grosvenor Street, London, ON N6A 4V2 Canada; Department of Microbiology & Immunology, University of Western Ontario, London, ON N6A 5C1 Canada; Perinatal and Women’s Health, London Health Sciences Centre, London, ON N6A 4L6 Canada; Department of Biochemistry, University of Western Ontario, London, ON N6A 5C1 Canada

**Keywords:** Human milk, Milk microbiota, Factors affecting the milk microbiota

## Abstract

**Background:**

Human milk is an important source of bacteria for the developing infant and has been shown to influence the bacterial composition of the neonate, which in turn can affect disease risk later in life. Very little is known about what factors shape the human milk microbiome. The goal of the present study was to examine the milk microbiota from a range of women who delivered vaginally or by caesarean (C) section, who gave birth to males or females, at term or preterm.

**Methods:**

Milk was collected from 39 Caucasian Canadian women, and microbial profiles were analyzed by 16S ribosomal RNA (rRNA) sequencing using the Illumina platform.

**Results:**

A diverse community of milk bacteria was found with the most dominant phyla being Proteobacteria and Firmicutes and at the genus level, *Staphylococcus*, *Pseudomonas*, *Streptococcus* and *Lactobacillus*. Comparison of bacterial profiles between preterm and term births, C section (elective and non-elective) and vaginal deliveries, and male and female infants showed no statistically significant differences.

**Conclusions:**

The study revealed the diverse bacterial types transferred to newborns. We postulate that there may be a fail-safe mechanism whereby the mother is “ready” to pass along her bacterial imprint irrespective of when and how the baby is born.

**Electronic supplementary material:**

The online version of this article (doi:10.1186/s40168-015-0145-y) contains supplementary material, which is available to authorized users.

## Background

With the incidence of various non-infectious diseases on the rise, there is much interest in the developmental origins of health and disease and the potential role of early life feeding practices in modulating these outcomes. Breast-fed infants have been shown to be better protected than formula-fed infants against necrotizing enterocolitis and diarrhoea, allergy and asthma, inflammatory bowel disease, type 1 and type II diabetes, obesity and cardiovascular disease [1, 2]. In addition to immune protection and bioactive compounds being conveyed through maternal milk, a possible protective role of bacteria has been suggested. Lower than average levels of *Bifidobacterium* in human milk correlate with low levels of *Bifidobacterium* in the neonatal gut [3], allowing for higher than normal levels of *Bacteroides* to be established [4]. These high levels of *Bacteroides* early in life have been associated with an increased risk of asthma and obesity later in life [5–7]. Indeed, efforts to manipulate the microbiota of formula-fed infants through probiotic supplementation have resulted in protection against some of the above diseases, comparable to that observed for breast-fed infants [8–10].

Differences exist in bioactive components, macronutrients, cytokines, enzymes, proteins and immunological factors between preterm and term milk and milk from mothers giving birth by vaginal and caesarean deliveries [11–16]. As well, the energy content differs in milk depending on gender of the newborn, with breast milk from mothers who give birth to sons having more fat content than that of daughters [17, 18].

We hypothesized that physiological or hormonal triggers that influence milk composition might also support different bacterial genera. Thus, we studied human milk samples from women giving birth at different stages of gestation, by vaginal or caesarean delivery, and examined whether gender of the newborn also affected the microbiota profiles.

## Results

**Fig. 1:**
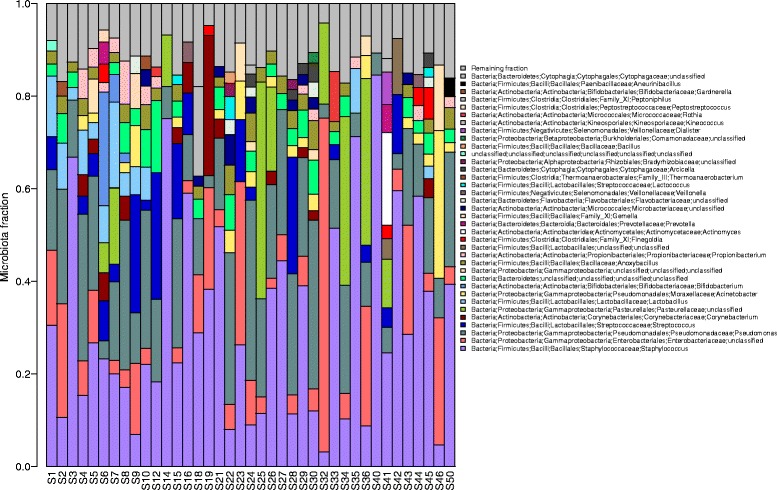
Breast milk microbiota in 39 Canadian women identified by 16S rRNA amplicon sequencing. The relative abundances of bacterial genera in different human milk samples were visualized by *bar plots*. Each bar represents a subject and each *coloured box* a bacterial taxon. The height of a coloured box represents the relative abundance of that organism within the sample. Taxa present in less than 2 % abundance in a given sample are displayed in the “remaining fraction” at the top of the graph (*grey boxes*). As shown by the bar plots, a variety of bacteria were detected in breast milk. The legend is read from bottom to top, with the bottom organism on the legend corresponding to the bottom coloured box on the bar plot

**Fig. 2:**
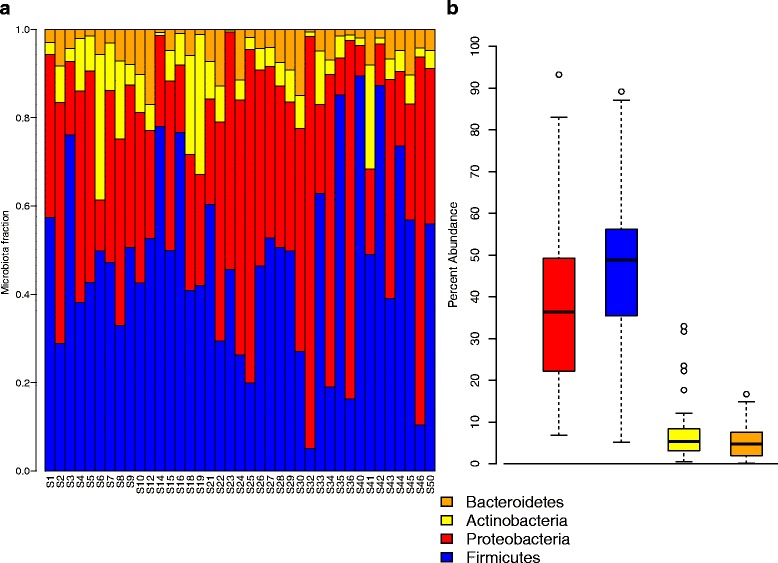
Percent abundances of bacterial phyla in breast milk identified by 16S rRNA sequencing. **a** The relative abundances of different phyla in different breast milk samples were visualized by *bar plots*. **b** *Box plots* of the four phyla identified in breast milk. The box signifies the 75 % (*upper*) and 25 % (*lower*) quartiles and thus shows where 50 % of the samples lie. The *black line* inside the box represents the median. The *whiskers* represent the lowest datum still within 1.5 interquartile range (IQR) of the lower quartile and the highest datum still within 1.5 IQR of the upper quartile. Outliers are shown with *open circles*. As shown, four phyla are represented in breast milk and present in all samples, with Proteobacteria and Firmicutes being the most abundant

Characterization of the milk microbiota in 39 Canadian women showed that despite diverse clinical parameters, almost all had high abundances of *Staphylococcus*, *Enterobacteriaceae* and *Pseudomonas* in their milk (Fig. 1) (Additional file 1: Table S1). Overall, the top 6 most abundant taxa represented in milk were *Staphylococcus* (31 %), *Enterobacteriaceae* (10 %), *Pseudomonas* (17 %), *Streptococcus* (5 %) and *Lactobacillus* (3 %) (Additional file 2: Figure S1). Out of the 47 genera detected, half (51 %) were present in all samples (Additional file 3: Table S2). At the phylum level, Proteobacteria and Firmicutes made up the highest proportion of phyla in all samples with Actinobacteria and Bacteroidetes also present but at lower levels (Fig. 2).

**Fig. 3:**
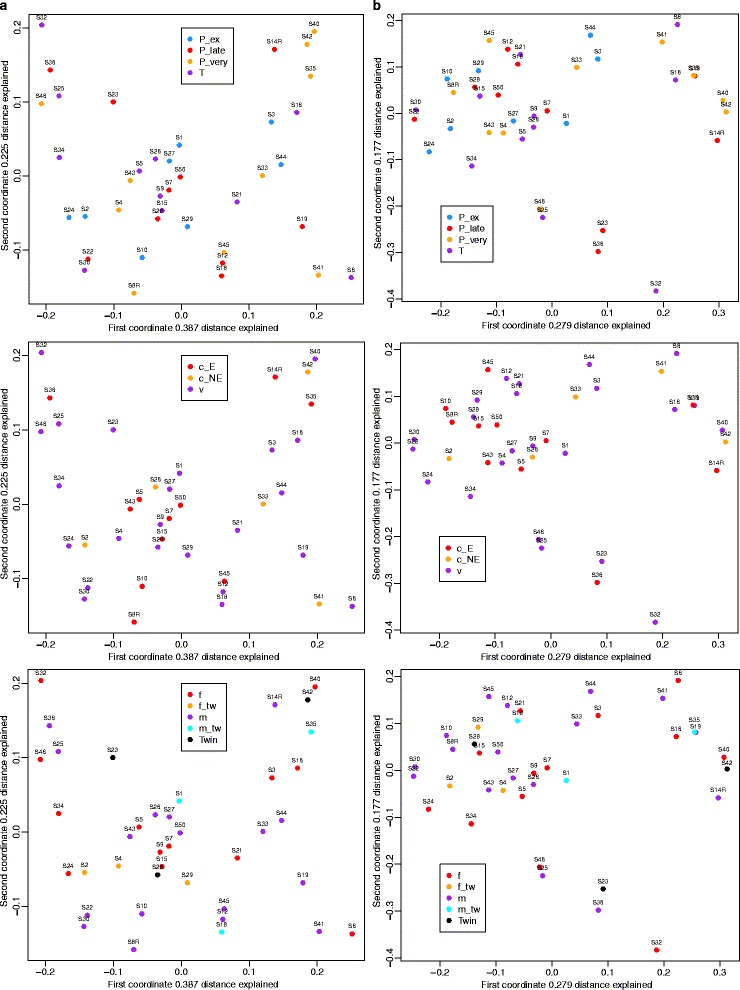
Principal coordinate analysis (PCoA) plots comparing bacterial profiles based on gestation, mode of delivery and gender. PCoA plots based on **a** weighted UniFrac distances or **b** generalized UniFrac distances at alpha 0.5. Each sample, represented by a *coloured circle*, is plotted on this two-dimensional, two-axis plane with the first two components plotted. Samples (*points*) that cluster together are more similar in biota composition and abundance. As shown by the plot, the lack of distinct clustering between groups, for gestation (1st row), mode of delivery (2nd row) and gender (3rd row), indicate that no bacterial differences exist between preterm and term samples, caesarean and vaginal delivery samples, and male and female samples. PERMANOVA (*p* < 0.5). P_ex = extremely premature (gestation <28 weeks); P_very = very premature (gestation 29–32 weeks); P_late = late premature (gestation 33–36 weeks); T = term (gestation >37 weeks); “c_E” = elective caesarean delivery; “c_NE” = non-elective C section; “v” = vaginal delivery; “m” = male child; “f” = female child; “m_tw” = twins both male; “f_tw” = twins both female; “Twin” = male and female twins

No differences were detected in microbial profiles based on gestation, mode of delivery or gender of the child, using both weighted (Fig. 3a) and un-weighted UniFrac distances (data not shown). The above two matrices, however, assign a large emphasis on either rare or highly abundant taxa, so compositional changes that occur in moderately abundant lineages may be overlooked [19]. For this reason, we also analyzed the data using generalized UniFrac at an alpha of 0.5, which overcomes this problem [19]. As with UniFrac, no differences were observed (Fig. 3b), but there was separation of 13 samples, forming 2 distinct clusters, which could not be explained by any of the other metadata that was collected (Additional file 4: Figure S2) (Additional file 1: Table S1). To further confirm the above results, we also utilized the Bray-Curtis dissimilarity metric, which does not take into account phylogenetic relatedness of the biological community, as does UniFrac. As expected, no differences were observed (Additional file 5: Figure S3).

The dataset was also analyzed using ALDEx2 [41] to examine whether specific taxa were differentially expressed based on gestation, mode of delivery or gender. Again, no differences were detected at the genus (Additional file 6: Table S3), family, class or phyla levels (data not shown).

Of interest, the abundance profiles clearly showed that mothers are transmitting very different bacterial profiles to newborns. For example, the milk of subject 40 contained over 80 % abundance of staphylococci, whereas that of subject 25 comprised mostly Gram-negative organisms, especially *Pseudomonas*.

## Discussion

This study revealed a range of bacterial genera in human milk, consistent with previous studies [20–22]. Interestingly, even when a baby was born extremely prematurely (subject S24), the mother’s milk was similar in composition to that of a woman giving birth at full term (subject S30). It can be speculated that this might be a fail-safe mechanism whereby the mother is “ready” to pass along her bacterial imprint irrespective of when the baby is born. If so, the microbiota would appear to be recalcitrant to late pregnancy hormonal and inflammatory changes, which could indicate an evolutionary pressure protecting this niche for the baby’s benefit. Further studies on this concept are warranted, and cases where the milk profiles are very different or the outcome for the baby negative could be particularly insightful.

It was not surprising that milk from emergency caesarean (C) section deliveries (i.e. non-elective) did not differ from women who gave birth vaginally, as this decision is made at the time of labour, when the hormones and timing for birth have been initiated. We also did not see differences between non-elective C section deliveries and vaginal births which is in contrast to a study published by Cabrera-Rubio et al. in which they concluded that the human milk microbiome is shaped by mode of delivery [23]. However, we do not believe that the authors adequately proved this claim. In their analysis, the milk samples collected were from both obese and normal weight women with no indication of the proportion in each group. Since it was shown in that same paper that body mass index influences the milk microbiota, the subject’s weight could have been a confounding factor responsible for the observed differences [23]. In addition, the author’s claim that the milk microbiota is influenced by mode of delivery was based solely on visual observations of bar plots (which were not very distinct between the two groups), with no principal coordinate analysis (PCoA) plots or statistical analyses to support this claim. These conflicting results between our group and that of Cabrera-Rubio highlight the need for future studies with larger sample sizes and inclusion of women from various demographics.

Because of the multivariate nature of the data, differences, if they are present, may have been masked by the different variables confounding each other. Thus, a larger sample size allowing for linear regression analysis would strengthen our claims. However, there is the possibility that no matter the sample size, differences will only be apparent when examined at the level of the individual. Schwarzberg et al. [24] showed that treatment for periodontitis had no effects on microbial profiles when compared to controls. However, when pre- and post-treatment effects for each individual were compared, significant changes in bacterial composition were observed. The authors thus emphasized the importance of comparing shifts from a personalized healthy state to a personalized disease state in order to truly understand biological changes [24].

Proteobacteria and Firmicutes were the dominant phyla, consistent with a previous high-throughput study performed in Switzerland and analyzed with a different sequencing platform (454 sequencing) [21].

The detection of *Staphylococcus*, as the most abundant organism, is likewise consistent with other studies [20–22]. *Staphylococcus* is more abundant in the gut of breast-fed newborns compared to formula-fed ones [25, 26], but their numbers start to decrease after the first week of life when oxygen has been consumed and an environment favourable for anaerobes is created [4, 27]. Unlike *Staphylococcus*, which is present in high abundance in at least the first week of life, Proteobacteria are never found in high numbers in the faeces of newborns, infants or Western adults [4, 28, 29]. Thus, an important question arises, as to what the evolutionary significance is of having such a diverse population of bacteria in milk, if only a select few seem to colonize during the development of the neonate. There are a few possibilities; firstly, persistent colonization is not always needed for beneficial effects, as transient exposure can be just as effective [30, 31]. Secondly, bacteria in milk may not have to be passed on from the mother to the child to exert their beneficial effects. Many of the protective factors in milk such as antibodies, immune cells, lactoferrin and beta defensins originate from the mammary gland and not from the blood [32]. With the ability of bacteria to regulate host gene expression, such as anti-microbials, and their ability to stimulate the immune system, the plethora of bacteria in breast milk could be inducing up-regulation of these protective factors in the breast that then get passed on in high concentrations to the neonate via milk.

From another perspective, there is the possibility that some of these milk microbes have either limited or no effects on the offspring but are present for the benefit of the mother, such as in the protection against mastitis. Mastitis is a painful inflammatory condition of the breast with the main causative agent being *Staphylococcus aureus*, and it has been shown that some milk commensals have the ability to prevent *S .aureus* growth and infection [33]. The same is true for a skin derived strain of *Propionibacterium acnes*, which prevents growth of *S. aureus* via its by-products of glycerol fermentation [34]. With the high abundance of glycerol present in milk, milk-derived strains of *Propionibacterium* may have similar growth-inhibiting properties, which would account for its presence in every milk sample collected.

*Lactobacillus* was present in high abundance in milk, so for those women who deliver by C section and thus do not transfer lactobacilli from the vagina to the infant, the milk could provide a means for these beneficial organisms to reach the infant gut.

## Conclusions

A diverse population of bacteria is present in breast milk dominated by the phyla Proteobacteria and Firmicutes and the taxa *Staphylococcus*, *Pseudomonas*, *Enterobacteriaceae*, *Streptococcus* and *Lactobacillus*. While no differences in microbial profiles were apparent based on gestation, mode of delivery or gender, more studies are still needed on what factors do influence bacterial communities in milk and how these changes impact neonatal and maternal health.

## Methods

### Milk collection and processing

A single milk sample (day 6 and onwards post-partum) was collected from 39 Caucasian Canadian women recruited from London, Ontario, and the surrounding area, representing a homogenous community. Even though the samples collected were from different days after birth, with some considered “transitional” and others “mature,” a Kendall’s tau correlation test showed no statistically significant correlation between operational taxonomic unit (OTU) relative abundances over time after a Benjamini-Hochberg correction for multiple hypothesis tests. This shows that it is acceptable to use all milk samples, regardless of when it was collected, in our analyses. Ethical approval was obtained from Western Research Ethics Board and Lawson Health Research Institute, London, Ontario, Canada. Subjects provided written consent for sample collection and subsequent analyses. Participants were excluded if they were suffering from mastitis and were/had been on antibiotics during lactation. Caesarean deliveries were classified as either (i) “non-elective”, if there were complications during labour, or (ii) “elective”, if they were planned in advance or if the health of the foetus and/or mother was at risk prior to labour.

Wearing sterile gloves, the women cleaned their nipple and surrounding area with sterile saline swabs to reduce the presence of skin bacteria. Milk was collected using a sterile HygieniKit Milk Collection System (Ameda, Buffalo Grove, IL, USA) attached to an electric breast pump. Between 5 and 15 ml of milk was pumped into a sterile tube and kept on ice until transfer to the laboratory, which occurred within 1 h of collection. Samples were aliquoted and stored at −20 °C until DNA extraction.

### DNA isolation

After thawing on ice, 2 ml of milk were spun down at 20,000×*g* for 10 min and the supernatant was discarded. The pellet was then homogenized in 1.4 ml of ASL buffer (QIAamp^®^ DNA Stool Kit, QIAGEN: Valencia, CA, USA) and 400 mg of 0.1-mm diameter zirconium-glass beads (BioSpec Products, Bartlesville, OK, USA). Mechanical and chemical lyses were performed by bead beading at 4800 rpm for 60 s, then 60 s on ice (repeated twice) using a mini-beadbeater-1 (BioSpec Products) and then incubated at 95 °C for 5 min. Subsequent procedures were performed using the QIAGEN QIAamp^®^ DNA Stool Kit according to the manufacturer’s protocol, with the exception of the last step in which the column was eluted with 120 μl of elution buffer. DNA was stored at −20 °C until further use.

A no template PCR control and a DNA extraction kit reagent control were sequenced alongside the samples. We observed that the taxon abundances in the controls were uncorrelated with the abundances in the experimental samples, and the distance between the controls and samples was large. Thus, we conclude that the controls had different profiles than that of the milk samples (Additional file 7: Figure S4).

### V6 16S rRNA gene sequencing

#### PCR amplification

The genomic DNA isolated from the clinical samples was amplified using barcoded primers that amplify the V6 hypervariable region of the 16S ribosomal RNA (rRNA) gene which is 70 base pairs long:

- V6-Forward: 5′ACACTCTTTCCCTACACGACGCTCTTCCGATCTnnnn(8)CWACGCGARGAACCTTACC 3′
- V6-Reverse: 5′CGGTCTCGGCATTCCTGCTGAAC CGCTCTTCCGATCTnnnn(8)ACRACACGAGCTGACGAC 3′

nnnn indicates four randomly incorporated nucleotides and “8” represents a specific sample barcode sequence. The PCR was carried out in a 42 μl reaction containing 2 μl of DNA template (or nuclease-free water as a negative control), 0.15 μg/μl bovine serum albumin, 20 μl of 2X GoTaq hot start colourless master mix (Promega) and 10 μl of each primer (initial concentration 3.2 pmol/μl). Thermal cycling was carried out in an Eppendorf Mastercycler under the following conditions: initial denaturation at 95 °C for 2 min followed by 25 cycles of 95 °C for 1 min, 55 °C for 1 min, and 72 °C for 1 min. After amplification, the DNA concentration was measured with the Qubit^®^ 2.0 Fluorometer (Invitrogen) using the broad range assay. Equimolar amounts of each PCR product were then pooled together and purified using the QIAquick PCR purification kit (QIAGEN). The pooled PCR purified sample was then paired end sequenced on the Illumina MiSeq platform using a 150-cycle kit with a 2 × 80 run at the London Regional Genomics Centre, London, ON, Canada, following standard operating procedures.

### Sequence processing and taxonomic assignment

Custom Perl and Bash scripts were used to de-multiplex the reads and assign barcoded reads to individual samples. Multiple layers of filtering were employed: (i) Paired end sequences were overlapped with Pandaseq, allowing zero mismatch in the overlapped reads; (ii) Reads were kept if the sequence included a perfect match to the V6 16S rRNA gene primers; (iii) Barcodes were 8mers with an edit distance of >4, and reads were kept if the sequence were a perfect match to the barcode; (iv) Reads were clustered by 97 % identity into OTUs using the Uclust algorithm of USEARCH v7 [35] which has a de novo chimera filter built into it; and (v) All singleton OTUs were discarded, and those that represented ≥1 % of the reads in at least one sample were kept.

Taxonomic assignments for each OTU were made by extracting the best hits from the SILVA database [36] and then manually verified using the Ribosomal Database Project (RDP) SeqMatch tool (rdp.cme.msu.edu) and by BLAST against the Green genes database (greengenes.lbl.gov). Taxonomy was assigned based on hits with the highest percentage identities and coverage. If multiple hits fulfilled this criterion, classification was re-assigned to a higher common taxonomy. A summary of each OTU classification and its sequence is shown in Additional file 8: Table S4. The raw sequencing reads generated in this study have been deposited to the NCBI Short Read Archive (SRA) database accession # SRP 064311.

### Data analysis

Weighted and un-weighted UniFrac distances [37] were calculated in QIIME [38] by using a tree of OTU sequences built with FASTTREE [39] based on an OTU sequence alignment made with MUSCLE [40]. The QIIME pipeline was also used to generate PCoA plots to visualize the Bray-Curtis dissimilarity. Changes in the microbial community composition were also analyzed by calculating the generalized UniFrac distance (alpha = 0.5, rooted phylogenetic tree) using the GUniFrac package in R, version 3.1.2 [19]. PERMANOVA was used to test for statistical significance between the groups using 10,000 permutations (QIIME package). Bar plots, box plots, stripcharts, PCoA plots and *k* means clustering analysis were all generated in R (http://www.R-project.org/).

The ALDEx R package version 2 [41] was used to compare genera, class, family and phyla between preterm and term milk; caesarean and vaginal deliveries, and male and female children. Values reported in this manuscript represent the expected values of 128 Dirichlet Monte Carlo instances. A value of zero indicates that organism abundance is equal to the geometric mean abundance. Thus, organisms more abundant than the mean will have positive values, and those less abundant than the mean will have negative values. Base 2 was used for the logarithm, so differences between values represent fold changes. Significance (*p* < 0.05) was based on the Benjamini-Hochberg corrected *p* value of the Welch’s *t* test and the Wilcoxon test.

## Availability of supporting data

The data set supporting the results of this article are included within Additional file 1: Table S1; Additional file 2: Figure S1; Additional file 3: Table S2; Additional file 4: Figure S2; Additional file 5: Figure S3; Additional file 6: Table S3; Additional file 7: Figure S4; Additional file 8: Table S4.

## Additional files

Additional file 1: Table S1. Summary of clinical data collected from 39 Canadian women. (XLSX 41 kb) Additional file 2: Figure S1. Five most abundant genera in human milk. Each point on the graph represents a subject, which indicates the percent relative abundance of that genus within the sample. The line represents the mean for all samples within the group. (PDF 25 kb) Additional file 3: Table S2. Genera present in every milk sample. (XLSX 57 kb) Additional file 4: Figure S2. Principal coordinate analysis (PCoA) based on generalized UniFrac distances. Each sample, represented by a coloured circle, is plotted on this two-dimensional, two-axis plane with the first two components plotted. Samples (points) that cluster together are more similar in biota composition and abundance. GUniFrac, using an alpha of 0.5 (which is more sensitive to changes in moderately abundant taxa), was used to compare microbial profiles based on gestation, mode of delivery and gender. While no differences were seen based on these conditions, there were three distinct groups which could not be explained by any of the metadata collected (Table S1). The number of clusters was determined using the *k* means clustering analysis in R. (PDF 28 kb) Additional file 5: Figure S3. Bray-Curtis dissimilarity principal coordinate (PCoA) plots comparing bacterial profiles based on gestation, mode of delivery and gender. Each sample, represented by a coloured circle, is plotted on this 3D, three-axis plane. Samples (points) that cluster together are more similar in biota composition and abundance. As shown by the plot, the lack of distinct clustering between groups, for Gestation (1st row), mode of delivery (2nd row) and gender (3rd row), indicate that no bacterial differences exist between preterm and term samples, caesarean and vaginal delivery samples, and male and female samples. P_ex = extremely premature (gestation <28 weeks); P_very = very premature (gestation 29–32 weeks); P_late = late premature (gestation 33–36 weeks); T = term (gestation >37 weeks); “c” = caesarean delivery; “v” = vaginal delivery; “m” = male child; “f” = female child; “m_tw” = twins both male; “f_tw” = twins both female; “Twin” = male and female twins. (PDF 689 kb) Additional file 6: Table S3. Comparison of relative abundances of different genera detected in milk between preterm and term samples, caesarean and vaginal deliveries, and male and female child. The values reported for “rab.sample.” represents the base 2 logarithm of the relative abundance of a specific genus within a sample. “rab.win..” represents the base 2 logarithm of the median abundance of a specific genus in all samples within a group (i.e. preterm samples or term samples) relative to the geometric mean abundance, which has a value of 0. Thus, positive values are higher than the geometric mean and are thus more abundant than negative values, which are lower than the geometric mean. Significance (*p* < 0.05) was based on the expected Benjamini-Hochberg corrected *p* value of the Welch’s *t* test (we.eBH) or the Wilcoxon test (wi.eBH). we.ep and wi.ep represent the raw *p* value of the respective tests. Out of the 47 genera identified, none were significantly different under any condition. (XLSX 139 kb) Additional file 7: Figure S4. Principal components analysis (PCA) biplot comparing milk samples with controls. To verify that the microbiota observed in our milk samples was not due to background contamination from reagents in either the DNA extraction kit or from the PCR, a no template PCR control (“NTC1”) and a tube of PBS that was extracted alongside the milk samples (“reagent_control”) were sequenced. Data presented in the biplot are from centred log ratio transformed values [42]. As observed, the controls have a different microbial profile than the milk samples. (PDF 24 kb) Additional file 8: Table S4. Summary of taxonomic results and full length V6 16S rRNA sequence of each operational taxonomic unit (OTU). (XLSX 35 kb)
