# Supplementary figures and images for: Human milk microbiota profiles in relation to birthing method, gestation and infant gender
Source: Microbiome. 2016 Jan 6;4:1. doi: 10.1186/s40168-015-0145-y (PMC4702315; doi:10.1186/s40168-015-0145-y)

Percent abundance

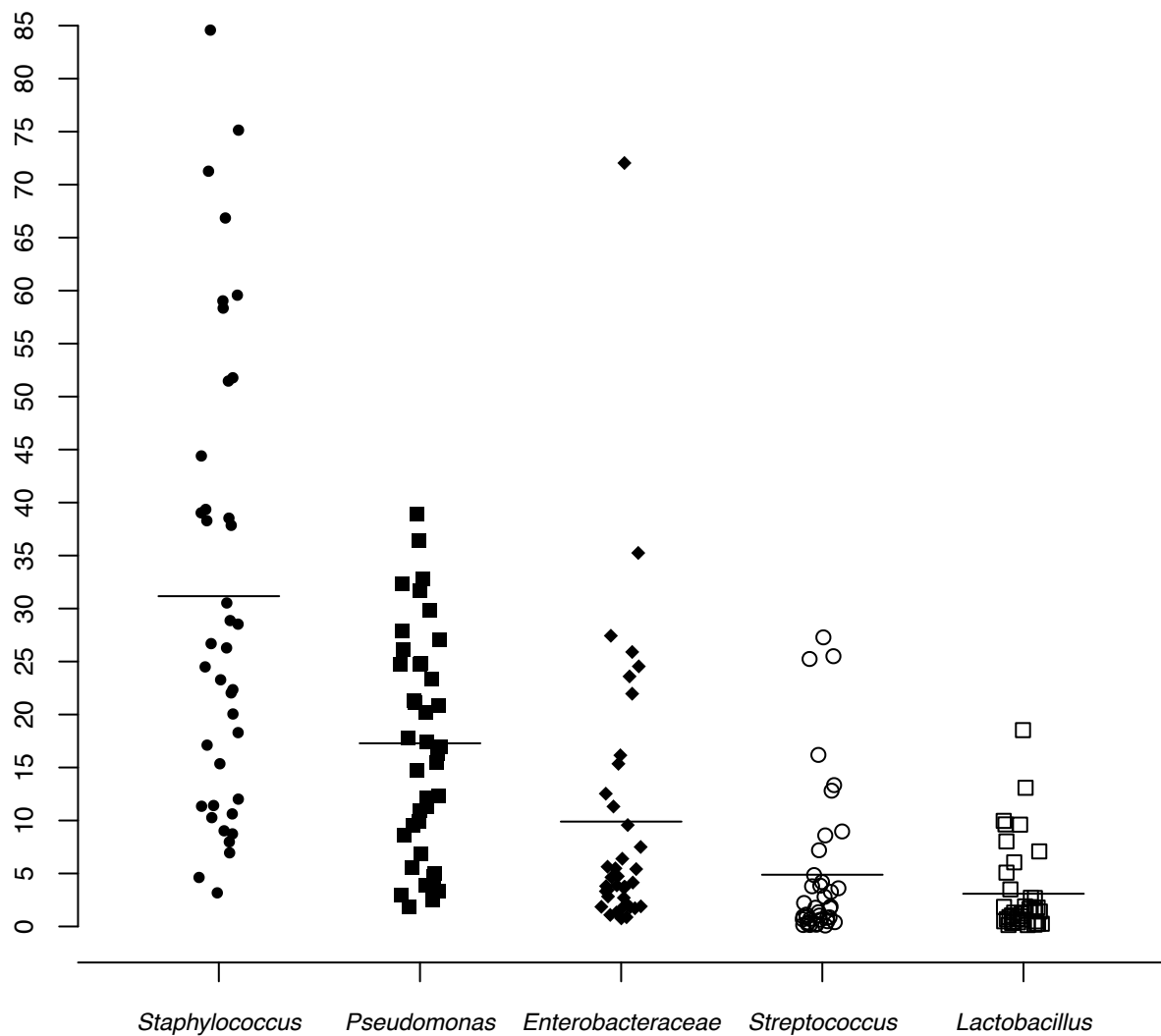

Supplement: Additional file 2: Figure S1. — Five most abundant genera in human milk. Each point on the graph represents a subject, which indicates the percent relative abundance of that genus within the sample. The line represents the mean for all samples within the group. (PDF 25 kb) [file 40168_2015_145_MOESM2_ESM.pdf]

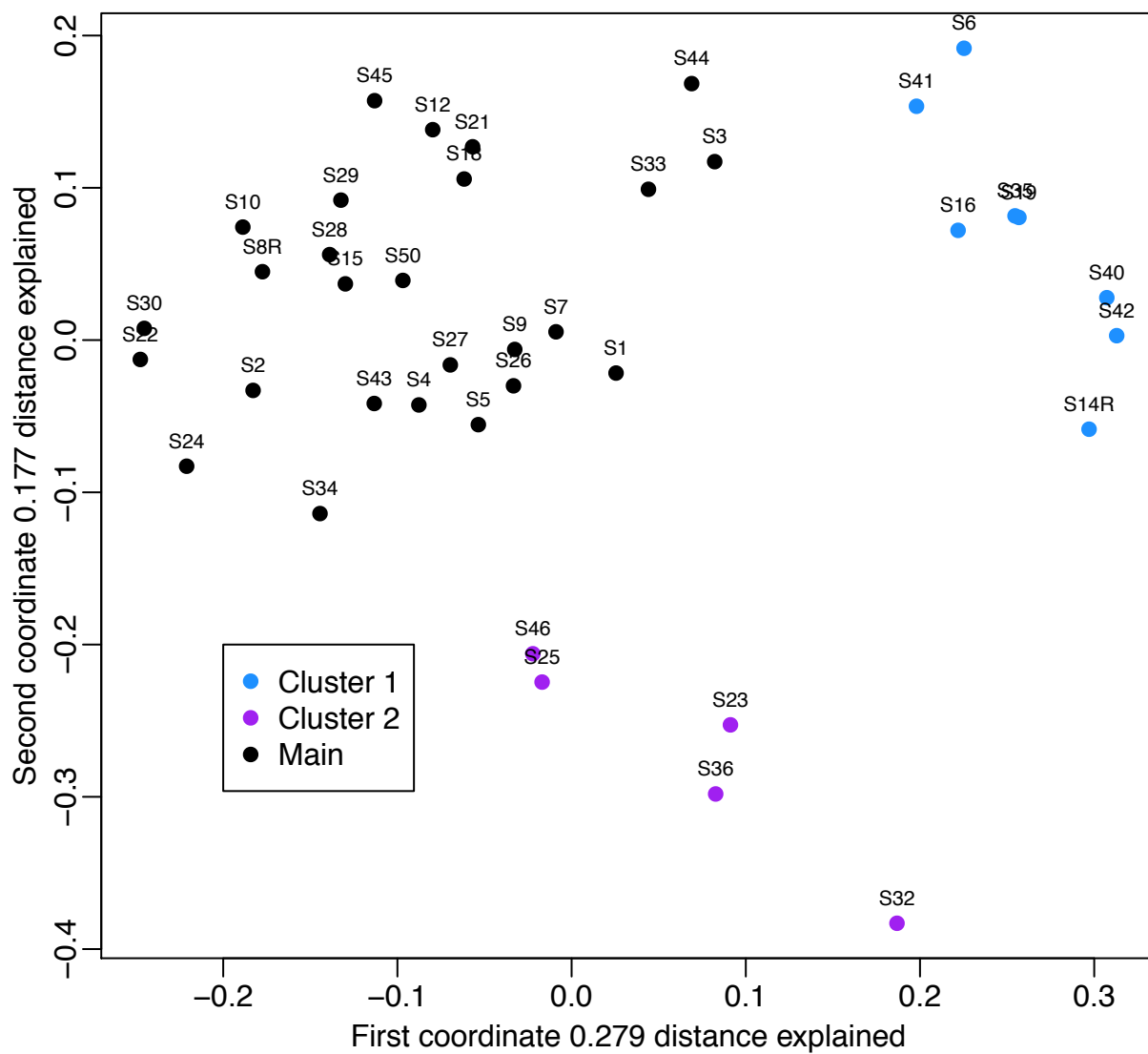

Supplement: Additional file 4: Figure S2. — Principal coordinate analysis (PCoA) based on generalized UniFrac distances. Each sample, represented by a coloured circle, is plotted on this two-dimensional, two-axis plane with the first two components plotted. Samples (points) that cluster together are more similar in biota composition and abundance. GUniFrac, using an alpha of 0.5 (which is more sensitive to changes in moderately abundant taxa), was used to compare microbial profiles based on gestation, mode of delivery and gender. While no differences were seen based on these conditions, there were three distinct groups which could not be explained by any of the metadata collected (Table S1). The number of clusters was determined using the k means clustering analysis in R. (PDF 28 kb) [file 40168_2015_145_MOESM4_ESM.pdf]

Gestation

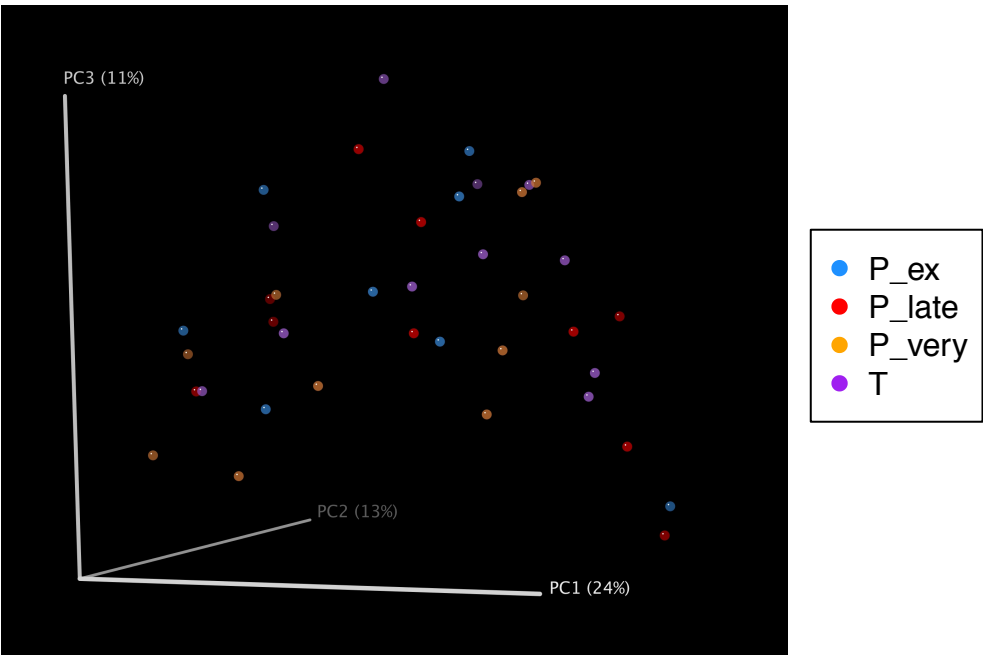

Delivery

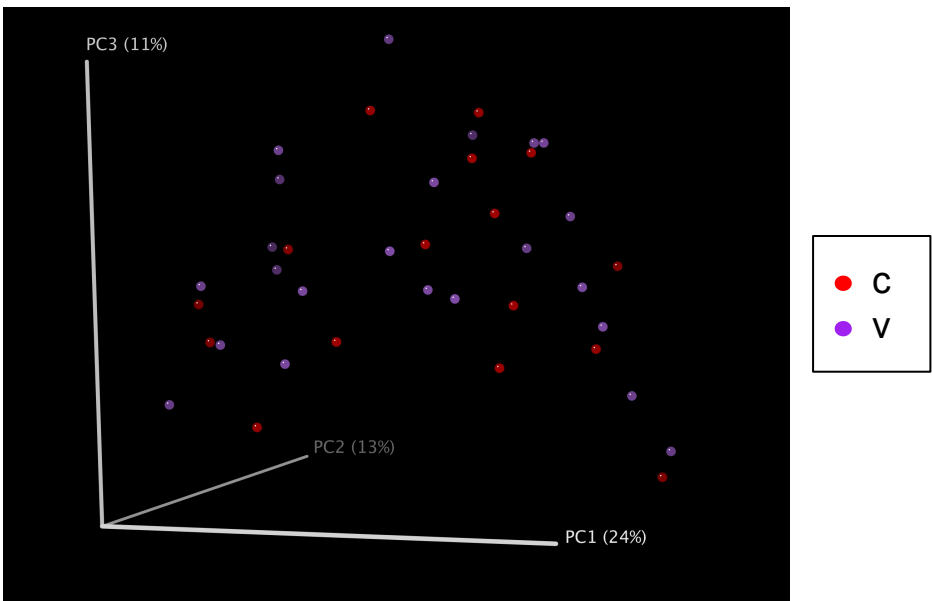

Gender

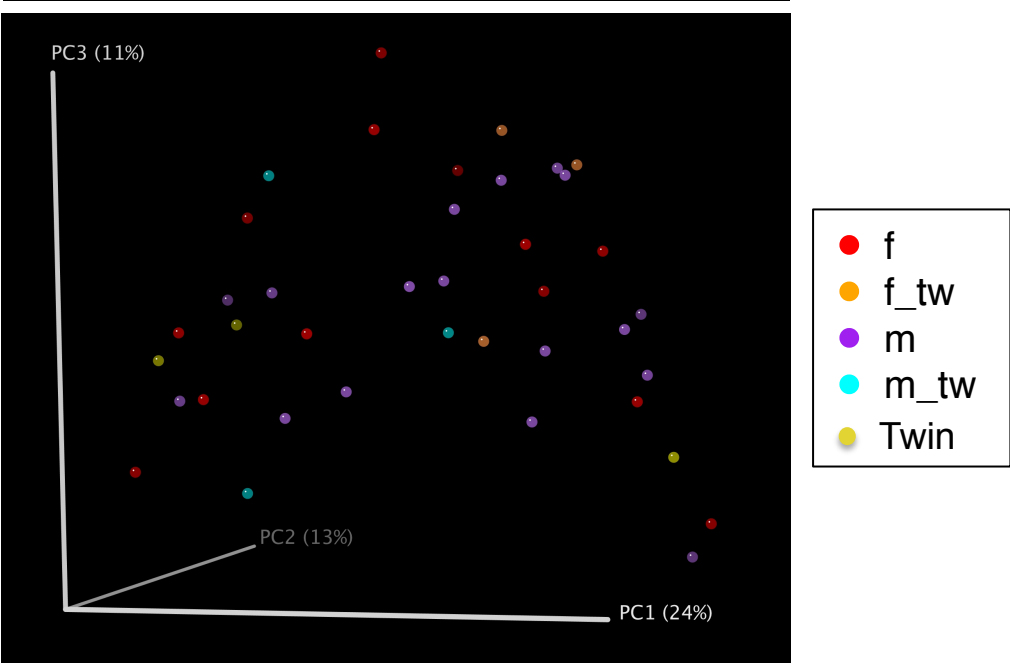

Supplement: Additional file 5: Figure S3. — Bray-Curtis dissimilarity principal coordinate (PCoA) plots comparing bacterial profiles based on gestation, mode of delivery and gender. Each sample, represented by a coloured circle, is plotted on this 3D, three-axis plane. Samples (points) that cluster together are more similar in biota composition and abundance. As shown by the plot, the lack of distinct clustering between groups, for Gestation (1st row), mode of delivery (2nd row) and gender (3rd row), indicate that no bacterial differences exist between preterm and term samples, caesarean and vaginal delivery samples, and male and female samples. P_ex = extremely premature (gestation <28 weeks); P_very = very premature (gestation 29–32 weeks); P_late = late premature (gestation 33–36 weeks); T = term (gestation >37 weeks); “c” = caesarean delivery; “v” = vaginal delivery; “m” = male child; “f” = female child; “m_tw” = twins both male; “f_tw” = twins both female; “Twin” = male and female twins. (PDF 689 kb) [file 40168_2015_145_MOESM5_ESM.pdf]

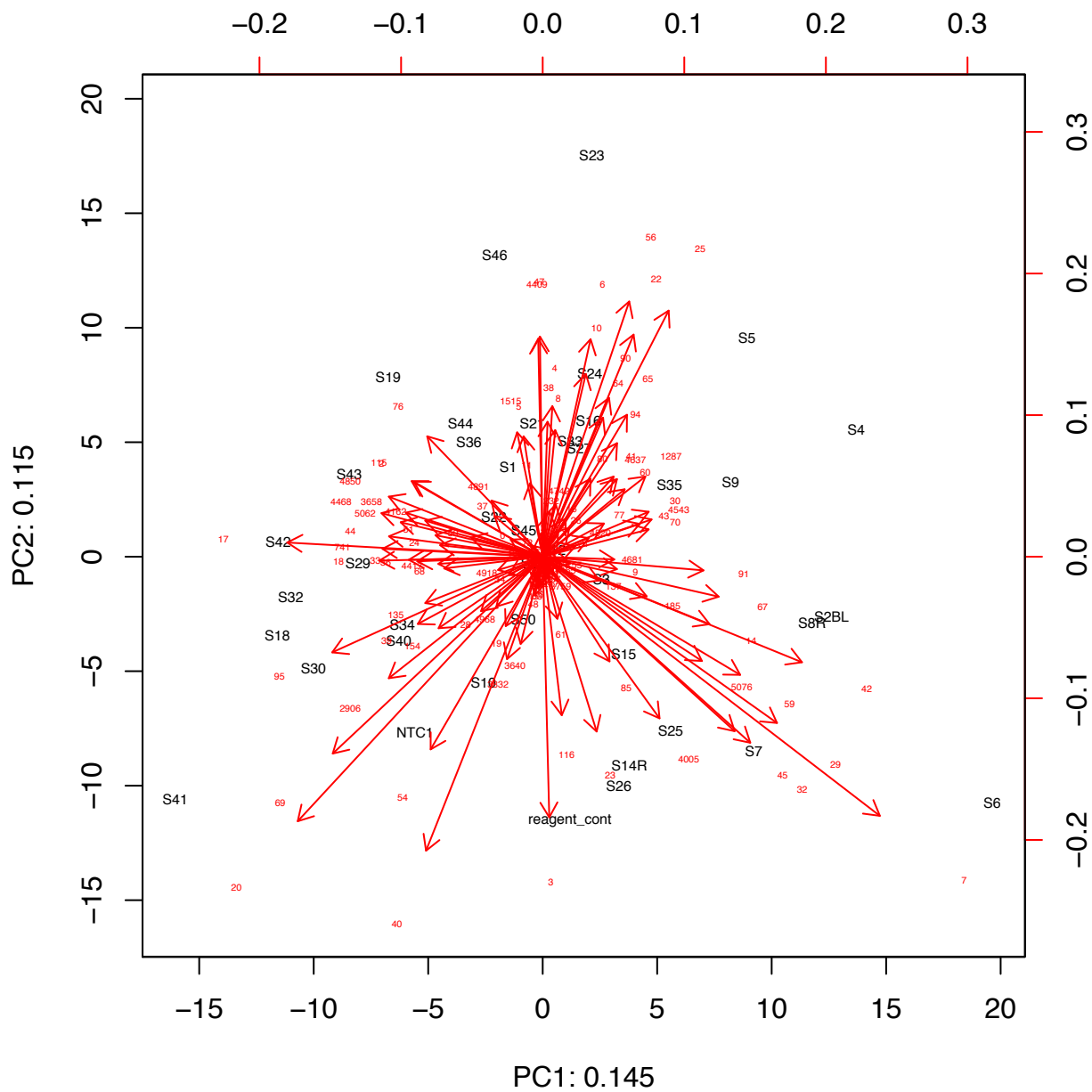

Supplement: Additional file 7: Figure S4. — Principal components analysis (PCA) biplot comparing milk samples with controls. To verify that the microbiota observed in our milk samples was not due to background contamination from reagents in either the DNA extraction kit or from the PCR, a no template PCR control (“NTC1”) and a tube of PBS that was extracted alongside the milk samples (“reagent_control”) were sequenced. Data presented in the biplot are from centred log ratio transformed values [42]. As observed, the controls have a different microbial profile than the milk samples. (PDF 24 kb) [file 40168_2015_145_MOESM7_ESM.pdf]
